# Assessing Frailty in the Older: The Role of Bite Force as an Independent Indicator

**DOI:** 10.3390/geriatrics10020040

**Published:** 2025-03-13

**Authors:** Luciano Maia Alves Ferreira, José Brito, Josie Resende Torres da Silva, Marcelo Lourenço da Silva, Maia e Maia Fischel e Andrade, André Júdice, José João Mendes, Vanessa Machado, João Thiago Botelho, Simone Cecílio Hallak Regalo

**Affiliations:** 1 Neuromodulation and Pain Unit (NeuroPain), Egas Moniz School of Health and Science, 2825-511 Almada, Portugal; 2 Egas Moniz Interdisciplinary Research Centre (CiiEM), Egas Moniz School of Health and Sciences, 2825-511 Almada, Portugal; 3 Laboratory of Neurosciences, Neuromodulation and Pain Studies (LANNED), Federal University of Alfenas, 37130-001 Alfenas, Minas Gerais, Brazil; 4 Faculty of Dentistry of Ribeirão Preto, University of Sao Paulo (FORP/USP), 14040-904 Ribeirão Preto, São Paulo, Brazil

**Keywords:** bite force, grip strength, fragility, oral health, phenotype

## Abstract

**Background:** This study investigates the relationship between bite force and grip strength as indicators of frailty in older adults. Frailty syndrome, characterized by increased vulnerability to adverse health outcomes, poses significant challenges in geriatric care. **Objectives:** This research builds on previous findings linking oral health to frailty risk, emphasizing the need for targeted interventions. **Methods:** A total of 59 older participants, aged 60 years and older, were enrolled in this cross-sectional study conducted at the Egas Moniz School of Health and Science. The participants underwent assessments of bite force using an electric dynamometer and grip strength using a specialized device. Body composition was also measured using bioelectrical impedance analysis (BIA). **Results:** Statistical analysis revealed a significant positive correlation between bite force and grip strength, even after adjusting for age and body mass index (BMI). Age was significantly correlated with bite and grip force (*p* < 0.05), while BMI was correlated only with handgrip force but not with bite force (coefficient = −0.047, *p* = 0.737). Notably, bite force was found to be independent of BMI, unlike grip strength, which is generally influenced by body composition. This independence highlights the potential of bite force as a reliable and distinct marker for frailty that is not confounded by BMI-related factors. This study highlights the importance of oral health in maintaining overall well-being in older adults. Reduced bite force may indicate an increased risk of frailty, which can lead to malnutrition and decreased quality of life. These findings suggest that integrating bite force measurements into clinical assessments may improve the assessment of frailty and inform interventions aimed at improving health outcomes in the older population. **Conclusions:** This research provides new insights into the association between bite force and grip strength, emphasizing the unique value of bite force as an independent marker of frailty. It advocates for further studies to explore its role in geriatric care strategies.

## 1. Introduction

The aging of the population in Europe has come to the fore due to its extensive and rapid occurrence. This situation poses considerable challenges, particularly in the acquisition of the necessary resources for the implementation of public policies that favor active aging and counteract the multiple common comorbidities that exist in these populations [1]. Such health problems contribute to the development of geriatric syndromes, which in turn can hinder the autonomy and independence of the older, known as frailty [2]. This can result in disability, vulnerability, institutionalization, and ultimately premature death [3,4,5].

Frailty is closely associated with greater vulnerability to negative health consequences and increased susceptibility to adverse physical outcomes, especially in older populations of various age groups. Although the incidence of frailty with age is not necessarily a direct result of the natural aging process itself, it remains a critical factor to address [6]. Several criteria have been proposed to identify frailty, often assessed by the accumulation of physiological and/or functional deficits [7,8].

Identifying vulnerabilities and limitations can facilitate the recognition of frailty phenotypes, which allows for more targeted and multidimensional interventions. Frailty has been defined as a clinical syndrome characterized by the presence of three or more of the following criteria: involuntary weight loss (of 4.5 kg or more in the previous year), self-reported exhaustion, weakness indicated by grip strength, slow walking speed, and low levels of physical activity [9].

These characteristics both result from and contribute to the accumulation of subclinical conditions, such as acute and chronic diseases, as well as physical, social, and behavioral risk factors. Although many negative health outcomes in older adults can occur independently, there is a consensus that frailty is a clinical syndrome characterized by the association and interdependence of several phenotypes, including muscle weakness, bone fragility, low body mass index, risk of falls, susceptibility to trauma, high risk of infections, fluctuations in blood pressure, and different degrees of impairment of physical abilities [10,11,12].

Bite force in older adults is a crucial component of masticatory function and tends to decrease with age. Studies indicate that maximum bite force gradually decreases throughout life, influenced by factors such as tooth loss, muscle changes, and health conditions. While young adults can exert a bite force of up to 60 kg, this capacity decreases significantly in older individuals, especially after 65 years of age, affecting masticatory capacity, nutrition, and quality of life. Denture use, temporomandibular disorders, and neurological diseases such as Parkinson’s also impact bite force. Measuring bite force with gnatodynamometers is essential for diagnosing and treating stomatognathic system disorders as well as guiding interventions aimed at improving chewing in the older [13].

In the medical field, frailty and oral health are evaluated through characteristics directly related to physical function. In Japan, Miura et al. had already studied this phenomenon and added bite force and oral health to frailty in the older, with emphasis on specific data obtained by the Dental Prescale system [14]. They reported the following conclusions and data: bite force is essential for maintaining a healthy lifestyle and eating healthy foods; the median bite force in healthy older adults varies between the sexes (men have greater force than women); frail older adults have significantly lower bite force compared with healthy older adults; bite force correlates with the number of teeth present (suggesting that a decrease in bite force may occur before tooth loss in frail older adults); and finally, despite the reduction in bite force among frail older adults, oral health care support for older adults is insufficient. The text already indicated that with the rapid increase in the older population in Japan, the importance of considering guidelines to improve chewing and oral health care should increase in the future [15,16].

In recent years, the understanding of frailty in the older has evolved, with a growing recognition of the multifaceted nature of this clinical syndrome [7]. While earlier studies have predominantly focused on grip strength as a primary indicator of frailty [9], our research introduces bite force as a significant marker of overall muscle function. This change in focus is fundamental, as it highlights the importance of oral health in the broader context of geriatric care.

The objective of this study was to explore whether bite force correlates with handgrip strength independently in addition to clinically highlighting the importance of bite force in the health and quality of life of older adults, especially in the context of frailty, suggesting the need for adequate oral health care to meet the growing needs of the older population.

## 2. Materials and Methods

### 2.1. Design and Configuration

This study adheres to the recommendations of the Strengthening the Reporting of Observational Studies in Epidemiology (STROBE) guideline checklist of cross-sectional studies [17]. This study was carried out at the Egas Moniz School of Health and Science between January and May 2024. Older people attending clinics at the Monte da Caparica campus (Almada, Portugal) were invited to participate in this study.

This study was approved by the Ethics Committee of Egas Moniz (ID 1104/2022, 30 June 2022). The participants initially signed an informed consent form containing all information related to this study, including privacy and confidentiality terms, the risks and benefits of this study, and the possibility of withdrawal at any time.

### 2.2. Eligibility Criteria

The participants were required to be 60 years of age or older, have functional teeth (at least 20 teeth), and have a state of oral cognition and functioning that is suitable for independent individuals. The exclusion criteria included having more than 20 missing teeth (for any reason), being completely edentulous, and having any clinical diagnosis of dementia-related neurological pathology. The participants were required to be 60 years of age or older, have functional teeth (at least 20 teeth), and present a state of oral cognition and functioning for independent individuals. The exclusion criteria included having more than 20 teeth missing for any reason, being completely edentulous, and having any clinical diagnosis of dementia-related neurological pathology.

### 2.3. Bite Force Measurement and Protocol

An electric dynamometer (EMG System^®^, São José dos Campos, São Paulo, Brazil) was used to measure the bite force. The dynamometer was covered with disposable material, and its mechanical components were sanitized and calibrated according to the manufacturer’s instructions.

The participants were instructed on how to perform the test. It was demonstrated by the tester, and each participant was able to try the device once before the test began. Once installed inside its support, each participant must remain seated and rested. They had a computer screen as biofeedback to monitor the result of the bite force classification. Data collection was performed in 3 full-force bite attempts lasting 10 s, with a rest interval of 1 min between each attempt.

Each participant, seated in an armless chair, would feel fully supported on the floor, with hips as far back as possible in the chair and the hips and knees positioned at approximately 90°.

The following instructions were given. This test will tell me his maximum bite force. When I say go, bite as hard as you can until I say stop. Before each attempt, I ask, ‘Are you ready?’ and then I’ll say ‘Go’. Stop immediately if you experience any unusual pain or discomfort at any time during the test [18].

### 2.4. Grip Strength Measurements and Protocol

Grip strength was measured using the K-Grip-Link^®^ (Kinvent, Montpellier, France), with values collected and recorded in kg/F. The mechanical parts were properly sanitized and calibrated according to the manufacturer’s instructions.

The participants were informed about the test and were given a tablet screen as biofeedback to monitor the results of the strength measurement. The data collection was performed in 3 series lasting 10 s, with a rest interval of 1 min, standardized by the K-Physio APP.

Each participant, seated in an armless chair, would feel fully supported on the floor, with hips as far back as possible in the chair and the hips and knees positioned at approximately 90°, as well as the shoulder adducted and rotated neutrally, the elbow flexed at 90° and in the middle prong (neutral), and the wrist between 15 and 30° extension (dorsiflexion) and 0 and 15° ulnar deviation.

The following instructions were given. This test will tell me your maximum Grip strength. When I say go, squeeze as hard as you can until I say stop. Before each attempt, I ask, ‘Are you ready?’ and then I’ll say ‘Go’. Stop immediately if you experience any unusual pain or discomfort at any time during the test [19].

### 2.5. Body Composition Measurements

Body composition was assessed by bioelectrical impedance analysis (BIA) with the ACCUNIQ BC310 model (Daejeon, Republic of Korea). Identity, age, and sex were entered into the software; height was measured using a stadiometer; and weight was recorded on the scale itself, which, after analysis, produced an automatic report with the body mass index (BMI) results. The participants were assessed in the morning, on an empty stomach, and were asked to abstain from physical exercise and drinks that contained any stimulant effect (coffee, energy drinks) in the last 24 h before the measurement.

The participants had to remove all metal objects (earrings, rings, belts) and wear as little clothing as possible, barefoot and without socks. They were each positioned standing on top of the scale, with their feet parallel, on top of the plantar sensors. The arms were close to the body, holding the palm sensors, and it was necessary to press the start button with both thumbs simultaneously for approximately 10 s (factory default). After completing the measurement, the older were assisted to step off the scale and instructed to get dressed and wear shoes throughout the assessment [20]. For convenience, we used BIA to estimate the BMI, while the remaining BIA data will be used in future studies.

### 2.6. Statistical Analysis

The association between bite and grip strength was assessed using the Pearson zero-order correlation coefficient. Partial correlation coefficients were also estimated to assess potential confounding effects due to age and BMI on the association between bite and grip strength. The correlation coefficients were estimated and tested after verifying the univariate normality of the variables involved using the Shapiro–Wilk normality test. All-subset regression was used to identify models with optimal performance, including bite force as the dependent variable and the four grip strengths and potential confounder of sex as predictors. This procedure removed the effect of multicollinearity between the bite force predictors and allowed control for confounding factors. The model comparison was based on Adjusted *R*^2^, the Akaike Information Criterion (AIC), and the Bayesian Information Criterion (BIC), with better model fit indicated by higher *R*^2^ values and by lower AIC and BIC values. The Gauss–Markov assumption for regression was checked using visual inspection of the plots of the residuals and the Durbin–Watson statistics for residual independence. Univariate and multivariate models were fitted to the data in group comparisons after model assumption checking. These statistical procedures were implemented with the IBM SPSS Statistics 29.0 software (Armonk, NY, USA) at a significance level of 5%.

## 3. Results Obtained

### 3.1. Characteristics of the Participants

Fifty-nine participants successfully completed this cross-sectional study, with no exclusions. Overall, the mean age was 78.6 years (±9.5), and 37.3% were institutionalized (Table 1).

The results of the univariate tests applied in the comparisons between the sexes show no differences between the groups for age and BMI and significantly taller and heavier men than women, as expected. The multivariate tests applied to the same groups detected no differences between the sexes for the bite force and significantly higher grip strength in men than in women. In view of these results, sex was included as a predictor in the models for bite force alongside grip force, ensuring that any sex-specific effects were appropriately accounted for in this analysis.

### 3.2. Bite and Grip Strength

Before evaluating the association between bite and grip strength, we explored age and BMI as potential confounding variables, as well as the correlations between the grip strength values, as multicollinearity between these forces could have an impact on the quantitative assessment of the abovementioned association. Pearson’s correlation coefficient was applied after verifying the univariate normality of the variables involved (Table 2). Age was significantly correlated with bite and grip strength (*p* < 0.05), while BMI was only correlated with handgrip force but not with bite force (coefficient = −0.047, *p* = 0.737). Moreover, and as expected, strong correlations were observed between the values of the strength of the grip, which may be the cause of the multicollinearity between those forces.

In these conditions, partial correlations between bite force and grip strength were calculated, controlling simultaneously for age and body mass index. It should be noted that the obtained partial correlation coefficients differ very little from the zero-order correlation coefficients presented in Table 1, which suggests that the effects of age and BMI on these correlations are not very relevant. In sum, altogether, these results show that bite force and grip strength show significant positive correlations. However, given the significant differences in grip strength between the sexes (Table 1), it is conceivable that those correlations might be affected by confounding effects due to sex.

**Figure 1:**
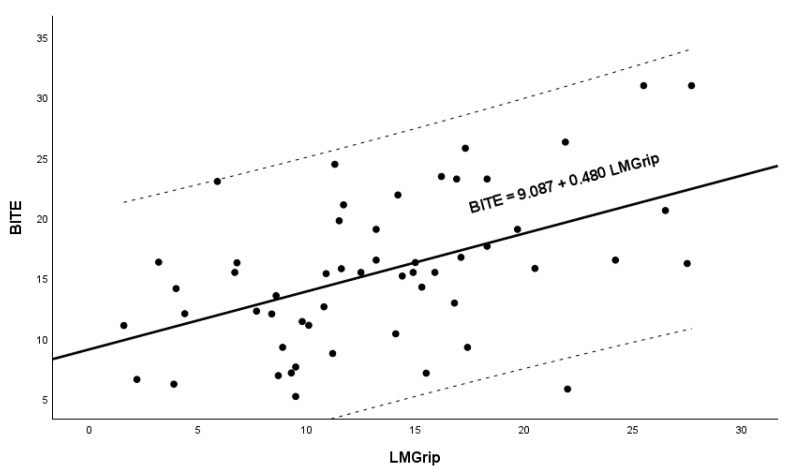
Relationship between bite force and Maximum Left Grip Force (LMGrip).

Therefore, linear regression models with bite force as the dependent variable and the four grip strengths and potential confounders by sex as independent variables were fitted to the data in an all-subset regression procedure to identify the models with optimal performance. This procedure accounted for the severe effects of multicollinearity between the grip strength values on the uncertainty of the model coefficients, which was confirmed by Variance Inflation Factor (VIF) values of greater than 30 observed in some models, while still controlling for the potential effects of confounders (Table 3). However, although some models with multiple predictors showed VIF values exceeding 30, those models were penalized in the selection process by lower adjusted *R*^2^ and higher BIC/AIC values, as well as by the failure of the tests of the coefficients to identify the significant predictors that are included in such models. The all-subset regression identified the following model (Figure 1), with a single predictor (LMGrip Force) as the predictive model of bite force with optimal performance indicated by higher *R*^2^ values and lower AIC and BIC values (Table 3): bite = 9.087 + 0.480 LMGrip. The final model selected, based on the adjusted *R*^2^, AIC, AICC, BIC and CAIC criteria, identified LMGrip as the sole predictor of bite force. Although LMGrip explains 23.3% of the variance in bite force, its high significance (*p* < 0.001) highlights its strong and reliable association with the dependent variable. Given that all the predictors represent measures of the same physiological nature (grip strength), this result supports LMGrip as the most relevant determinant of bite force among the predictors considered. Despite the moderate *R*^2^, this model remains valid and informative, as it provides a statistically sound basis for understanding the relationship between bite force and strength-related predictors while maintaining parsimony. Moreover, the final model, which includes a single predictor, is immune to multicollinearity concerns. It should be noted that the regression coefficient of LMGrip (0.480) remained stable after the inclusion of sex in the model (0.540), which demonstrates that the confounding effect due to this variable is minimal.

## 4. Discussion

This cross-sectional study aimed to investigate whether the measurement of grip strength, a component recognized as a frailty phenotype, could be correlated with bite force in older people. Overall, the correlation between grip strength and bite force was significant, with minimal confounding effects due to age, BMI, and gender. This suggests that bite force can serve as an indicator of frailty, complementing assessments of grip strength. By utilizing a specific sample of older adults with functional dentition, we provide new insights into the correlation between bite force and grip strength, suggesting that these measures may collectively inform frailty assessments. In addition, our findings reinforce the need for targeted interventions to improve oral health, which can play a vital role in improving the quality of life and nutritional statuses of older adults.

The relevance of these results reinforces the hypothesis of this study, seeking to characterize the measurement of bite force as a criterion (phenotype) of frailty as well as to demonstrate the importance of oral health in the older. This association is also important if related to the ease of the clinical application of this test, which is relatively fast and straightforward, like the handgrip protocols [19].

Unilateral grip strength is considered a very useful gold-standard protocol to identify the risk of frailty and mobility limitations in the older, but some important considerations should be highlighted about its ideal cut-off points, as they vary according to gender and body mass index (BMI). The cut-off points increase according to the BMI for men, while there is a specific threshold identified for women [21].

The literature also mentions specific cut-off values for grip strength associated with a higher probability of mobility limitation, of about 37 kg for men and 21 kg for women, but the accuracy of the risk identification is debated over specific BMI cut-off values. This approach improves the accuracy in men but not in women. Even so, it suggests that handgrip forces below the specific BMI cut-off value are associated with three times greater odds of mobility limitation in men and women.

Thus, this set of information proves that the assessment of handgrip force, considering gender and BMI, is a valuable tool in predicting the risk of mobility limitation, providing specific insights for different sociodemographic groups. However, it is important to note that the ideal cut-off points for assessing frailty components, such as grip strength, can vary based on factors such as gender and BMI. Future studies should explore the optimal cut-off values for bite force measurements and investigate their predictive value in identifying frailty risk in diverse demographic groups.

In this study, we observed a significant correlation between bite force and grip strength, particularly in males, where maximum and average right and left handgrip force were positively correlated with bite force. This finding suggests that bite force, like grip strength, may reflect overall muscle function and strength in older individuals.

The correlation between grip strength and bite force in our study supports the idea that individuals with higher overall muscle strength also tend to have stronger masticatory muscles. This relationship underlines the importance of maintaining muscle strength through physical activity and resistance training in older adults to preserve not only their mobility and independence but also their ability to perform vital functions such as chewing [21].

Men tend to have higher absolute muscle mass and strength compared to women, which may explain their superior performance on the grip strength tests. However, it is worth noting that despite these differences, women may also benefit from interventions aimed at maintaining or improving muscle strength as they age.

For Soraya and Parwanto [22], the relationship between the body mass index (BMI) and grip strength in the older is controversial, as studies have pointed out many discrepancies in the results. Some studies indicate a relationship between BMI and grip strength, while others do not show this relationship. In summary, the text highlights the lack of consensus in existing studies on the association between BMI and grip strength in the older and emphasizes the importance of future research to clarify this relationship and better understand how these factors are interconnected in the older population [23].

Our study provides an additional argument for discussion on this topic, demonstrating that there is a direct relationship between bite force and grip strength, with bite force being independent of BMI. This finding suggests that this type of assessment could be included as a criterion (phenotype) for frailty, providing an additional argument for discussions around frailty assessment methodologies and underlining the potential value of integrating bite force assessment into clinical practice as a complementary measure. In addition, this independence of body composition also makes bite force a safer phenotype.

The correlation between grip strength and bite force in our study supports the idea that individuals with higher overall muscle strength also tend to have stronger masticatory muscles. This relationship underlines the importance of maintaining muscle strength through physical activity and resistance training in older adults to preserve not only their mobility and independence but also their ability to perform vital functions such as chewing.

Iwasaki et al. demonstrated an association between maximal bite force (MMF), oral function, and frailty risk in older adults, where lower maximal bite force (MMF) would be associated with an increased risk of frailty in older adults [22]. Poor oral function should be identified as a factor that increases the risk of frailty in the older, and the relationship between oral function (OMF) and frailty is indicated as independent of the baseline characteristics of the participants. They also suggested that more research is needed to confirm these conclusions about the rate of development of frailty in older adults. In this study, over a 5-year follow-up period, 15.2% of participants developed frailty and significantly higher risk when they had lower maximal bite force. This highlights the multifaceted nature of frailty assessment and the potential to incorporate bite force measurement as a criterion for identifying and monitoring fragile individuals.

The results of this study have important implications for the health and nutrition of older adults. A reduction in bite force can have significant consequences for food intake, as it can limit the ability to chew hard or fibrous foods, such as fruits, vegetables, and meats, which are essential components of a healthy diet. Improper chewing due to decreased bite force can lead to a change in eating habits such as choosing industrialized and processed foods, poor digestion, reduced nutrient absorption, and ultimately malnutrition.

In addition, malnutrition and frailty often coexist in older adults, creating a vicious cycle that further compromises health and functional independence [9]. Frail individuals may struggle to consume the nutrients needed to maintain muscle mass and strength, leading to further declines in masticatory and overall muscle function. Interventions aimed at improving oral health, such as dentures or orthodontic devices, combined with nutritional support and physical rehabilitation can help break this cycle and improve overall health outcomes.

Thus, because bite force has been shown to correlate with grip strength, the assessment of masticatory function may provide a valuable, non-invasive method for screening for frailty in clinical settings. Since grip strength is already widely used to assess muscle function, incorporating bite force measurements could offer a more comprehensive assessment of an individual’s overall health.

Although this study provides valuable information, several limitations should be acknowledged. First, the sample size was relatively small, particularly for men, which may limit the generalizability of the results. Future studies with larger and more diverse populations are needed to confirm these findings and explore potential sex- and age-related differences in more detail.

Second, this study relied on BMI as a measure of body composition, following guidelines from the literature, which may not accurately reflect the distribution of muscle and fat mass in older adults. More accurate methods, such as dual-energy X-ray absorptiometry (DEXA), may provide a better understanding of how body composition influences both bite force and grip strength.

While grip strength is a widely accepted measure of muscle function, it does not capture the complexity of other muscle groups involved in activities of daily living, such as the muscles used for chewing. Oral frailty is characterized by several factors, including tooth loss, decreased bite force, chewing and swallowing problems, poor oral hygiene, and ill-fitting dentures [24]. The inclusion of oral frailty within the broader concept of frailty in older adults is an emerging topic in gerontological research, and assessment of bite force may be a simple and clinically relevant measure.

Future research could explore the relationship between bite force and other functional outcomes, such as mobility, balance, and cognitive function, to gain a more comprehensive understanding of how chewing force relates to overall health, a claim reinforced by the 2021 Lancet systematic review [25], which assessed the contribution of several oral health items to a possible operational definition of a new frailty phenotype, defined as a gradual age-related loss of oral function coupled with declines in cognitive and physical functions.

Recent studies have explored the relationship between oral health and frailty syndrome, highlighting the importance of considering oral aspects in the general assessment of older adults. For example, a cross-sectional observational study of 88 older adults found a clear correlation between oral and general health status between frail and non-frail groups [26], indicating a poorer perception of oral health for nutrition where oral problems can lead to malnutrition and involuntary weight loss, decreased tongue and masticatory muscle strength associated with frailty, and multimorbidity, as frail older adults tend to have higher numbers of systemic diseases and, for example, higher use of medications [27]. Therefore, longitudinal studies are needed to investigate how changes in bite force over time correlate with declines in muscle function, frailty, and nutritional status in older adults. Such studies may help identify critical points for intervention and inform strategies to preserve oral health and muscle strength as individuals age [28].

## 5. Conclusions

In conclusion, this study highlights the significant relationship between bite force and grip strength, particularly in older men, suggesting that bite force may serve as an indicator of overall muscle function. While age-related declines in bite force were evident, the lack of a clear correlation with BMI indicates that body composition factors such as fat and lean mass may play a more complex role in determining chewing force. These findings reinforce the importance of maintaining muscle strength through physical activities and interventions aimed at improving oral health in the older.

The implications for health and nutrition are substantial, as reduced bite force can contribute to malnutrition, frailty, and decreased quality of life in older adults. Assessment of bite force in clinical settings can provide valuable information for the early detection of frailty and guide interventions to preserve functional independence and well-being in older populations. Future research should focus on expanding sample sizes, using more accurate body composition measurements, and exploring the longitudinal effects of chewing force on overall health outcomes.

### Limitations

This study has some limitations, such as a small sample of 59 participants and a cross-sectional design, which only allowed the establishment of a relationship between bite force and grip force without allowing causal conclusions or temporal monitoring. In addition, this research was conducted in a single institution in Portugal, which limited the demographic diversity. Although these limitations have been acknowledged, it should be mentioned that the exclusion of individuals without teeth and with other oral health problems (common among people living with frailty) may have influenced the results.

## Figures and Tables

**Table 1:** Anthropometric and strength measurements by sex.

Variable	Men (*n* = 16)	Women (*n* = 43)	*p*-Value	Total
Age (years)	80 (7.89)	78.11 (10.11)	0.515	78.62 (9.53)
Height (cm)	164.1 (5.2)	153.7 (6.6)	<0.001	156.5 (7.8)
Weight (Kg)	75.1 (11)	66.4 (14.2)	0.028	68.8 (13.6)
BMI (kg/m^2^)	27.8 (4)	28.3 (5.7)	0.748	28.2 (5.2)
Bite (Kgf)	17.2 (7.7)	14.8 (5.7)	0.224	15.5 (6.3)
RMGrip (Kgf)	20 (8)	11.8 (4.9)	<0.001	14 (6.7)
LMGrip (Kgf)	18.7 (7.5)	11.7 (4.7)	<0.001	13.6 (6.3)
RAGrip (Kgf)	16.9 (6.6)	10 (4.6)	<0.001	11.9 (5.9)
LAGrip (Kgf)	16 (6.7)	10 (4.3)	<0.001	11.7 (5.6)

BMI—body mass index, Bite—maximum bite force, RMGrip—Maximum Right Grip Force, LMGrip—Maximum Left Grip Force, RAGrip—Average Right Grip Force, LAGrip—Average Left Grip Force.

**Table 2:** Correlation of age and BMI with bite and handgrip forces.

		Age	BMI	Bite	RMGrip	LMGrip	RAGrip
BMI	Correlation	−0.329					
	*p*	0.011					
Bite	Correlation	−0.321	−0.047				
	*p*	0.018	0.737				
RMGrip	Correlation	−0.340	0.316	0.448			
	*p*	0.008	0.015	<0.001			
LMGrip	Correlation	−0.414	0.295	0.497	0.911		
	*p*	0.001	0.023	<0.001	<0.001		
RAGrip	Correlation	−0.387	0.347	0.447	0.979	0.904	
	*p*	0.002	0.007	<0.001	<0.001	<0.001	
LAGrip	Correlation	−0.424	0.290	0.496	0.894	0.993	0.895
	*p*	<0.001	0.026	<0.001	<0.001	<0.001	<0.001

**Table 3:** Comparison between all-subset regression models. All models include an intercept and are significant at the 5% level of significance, except the model with sex as single predictor. For each model, significant predictors detected by the tests of coefficients are underlined. The selected model is indicated in bold.

Predictors in Model	*R*^2^ adj	AIC	AICC	BIC	CAIC	Max VIF
RMGrip, LMGrip, RAGrip, LAGrip	0.186	346.8	348.6	358.8	364.8	101.8
RMGrip, LMGrip, RAGrip	0.202	344.8	346.1	354.8	359.8	26.7
RMGrip, LMGrip, LAGrip	0.202	344.8	346.1	354.8	359.8	96.5
RMGrip, RAGrip, LAGrip	0.201	344.9	346.2	354.9	359.9	26.0
LMGrip, RAGrip, LAGrip	0.202	344.8	346.1	354.8	359.8	85.3
RMGrip, LMGrip	0.218	342.8	343.7	350.8	354.8	6.0
RMGrip, RAGrip	0.171	346.0	346.8	353.9	357.9	24.6
RMGrip, LAGrip	0.217	342.9	343.8	350.9	354.9	5.0
LMGrip, RAGrip	0.218	342.9	343.7	350.8	354.8	5.7
LMGrip, LAGrip	0.218	342.8	343.7	350.8	354.8	77.3
RAGrip, LAGrip	0.216	342.9	343.8	350.9	354.9	5.2
RMGrip	0.186	344.1	344.5	350.0	353.0	1
**LMGrip**	**0.233**	**340.9**	**341.3**	**346.8**	**349.8**	**1**
RAGrip	0.185	344.1	344.6	350.1	353.1	1
LAGrip	0.232	340.9	341.4	346.9	349.9	1
Sex, RMGrip, LMGrip, RAGrip, LAGrip	0.181	348.1	350.5	362.0	369.0	103.4
Sex, RMGrip, LMGrip, RAGrip	0.197	346.1	347.9	358.0	364.0	25.6
Sex, RMGrip, LMGrip, LAGrip	0.197	346.1	347.9	358.0	364.0	98.6
Sex, RMGrip, RAGrip, LAGrip	0.195	346.3	348.1	358.2	364.2	26.7
Sex, RMGrip, LMGrip	0.213	344.1	345.3	354.0	359.0	6.4
Sex, RMGrip, RAGrip	0.164	347.4	348.6	357.3	362.3	25.9
Sex, LMGrip, RAGrip	0.213	344.1	345.3	354.0	359.0	5.9
Sex, LMGrip, LAGrip	0.213	344.1	345.3	354.0	359.0	82.5
Sex, RAGrip, LAGrip	0.210	344.3	345.6	354.3	359.3	5.4
Sex, RMGrip	0.179	345.5	346.3	353.4	357.4	1.4
Sex, LMGrip	0.229	342.1	342.9	350.1	354.1	1.4
Sex, RAGrip	0.175	345.7	346.5	353.7	357.7	1.4
Sex, LAGrip	0.225	342.4	343.2	350.3	354.3	1.3
Sex	0.010	354.6	355.1	360.6	363.6	1

## Data Availability

The original contributions presented in this study are included in this article, other questions can be directed to the corresponding author.
